# Identification of Epidermal Pdx1 Expression Discloses Different Roles of Notch1 and Notch2 in Murine *Kras^G12D^*-Induced Skin Carcinogenesis *In Vivo*

**DOI:** 10.1371/journal.pone.0013578

**Published:** 2010-10-22

**Authors:** Pawel K. Mazur, Barbara M. Grüner, Hassan Nakhai, Bence Sipos, Ursula Zimber-Strobl, Lothar J. Strobl, Freddy Radtke, Roland M. Schmid, Jens T. Siveke

**Affiliations:** 1 2nd Department of Internal Medicine, Klinikum Rechts der Isar University Hospital, Technical University of Munich, Munich, Germany; 2 Department of Biological Sciences, University of Warwick, Coventry, United Kingdom; 3 Institute of Pathology, University Hospital, University of Tübingen, Tübingen, Germany; 4 Department of Gene Vectors, Helmholtz Zentrum, German Research Center for Environmental Health, Munich, Germany; 5 ISREC, Ecole Polytechnique Fédérale de Lausanne (EPFL SV ISREC), Lausanne, Switzerland; Bauer Research Foundation, United States of America

## Abstract

**Background:**

The Ras and Notch signaling pathways are frequently activated during development to control many diverse cellular processes and are often dysregulated during tumorigenesis. To study the role of Notch and oncogenic Kras signaling in a progenitor cell population, *Pdx1-Cre* mice were utilized to generate conditional oncogenic *Kras^G12D^* mice with ablation of *Notch1* and/or *Notch2*.

**Methodology/Principal Findings:**

Surprisingly, mice with activated *Kras^G12D^* and *Notch1* but not *Notch2* ablation developed skin papillomas progressing to squamous cell carcinoma providing evidence for *Pdx1* expression in the skin. Immunostaining and lineage tracing experiments indicate that PDX1 is present predominantly in the suprabasal layers of the epidermis and rarely in the basal layer. Further analysis of keratinocytes *in vitro* revealed differentiation-dependent expression of PDX1 in terminally differentiated keratinocytes. PDX1 expression was also increased during wound healing. Further analysis revealed that loss of Notch1 but not Notch2 is critical for skin tumor development. Reasons for this include distinct Notch expression with Notch1 in all layers and Notch2 in the suprabasal layer as well as distinctive p21 and β-catenin signaling inhibition capabilities.

**Conclusions/Significance:**

Our results provide strong evidence for epidermal expression of Pdx1 as of yet not identified function. In addition, this finding may be relevant for research using *Pdx1-Cre* transgenic strains. Additionally, our study confirms distinctive expression and functions of Notch1 and Notch2 in the skin supporting the importance of careful dissection of the contribution of individual Notch receptors.

## Introduction

Conditional tissue-specific modulation of genes using Cre/loxP recombination in genetically engineered mice provides an enormous leap forward to study gene function in detail yet requires detailed knowledge of gene regulation and expression patterns. For pancreatic targeting of genes, *Pdx1-Cre* mice are commonly used [1]–[3], in which Cre-recombinase is expressed under a 4.5 to 5.5 kb fragment of the *Pdx1* promoter. The transcription factor *Pdx1* (pancreas and duodenum homeobox gene 1) directs pancreatic cell formation, maintenance and function. *Pdx1* is expressed in the region of the endoderm that ultimately gives rise to stomach, pancreas and duodenum and its function is critical for posterior foregut development [4]. Postnatally, Pdx1 is mainly expressed in insulin-producing endocrine cells of the pancreas. Ablation of *Pdx1* results in defects of different cell types including malformations of the pylorus and duodenum, absence of Brunner's glands and reduced numbers of specific enteroendocrine cell types in the stomach and intestine. Loss of *Pdx1* function results in pancreatic agenesis, while heterozygous expression leads to defects in glucose homeostasis. *Pdx1*-deficient mice survive up to 6.5 days after birth, are severely dehydrated, have no fur and a delicate, cracking skin. [5]–[8]. Here, we report epidermal PDX1 expression observed due to an unexpected skin tumor formation in *Pdx1-Cre* mice with activation of oncogenic *Kras^G12D^* and loss of *Notch1* but not *Notch2*.

Notch proteins are evolutionarily conserved large transmembrane receptors, which upon ligand binding undergo proteolytic cleavage mediated by the γ-secretase-presenilin complex releasing the intracellular fragment (NIC). NIC is translocated to the nucleus where it binds and activates the mammalian repressor RBP-Jκ thereby regulating fetal and postnatal cell fate decisions and differentiation processes [9]. Notch receptors are expressed in the skin, although their precise functions remain uncertain (reviewed in [10], [11]). Gain- and loss-of-function studies have suggested various functions for Notch including proliferation control, differentiation switch of developing epidermis and formation of hair follicles [12]–[17]. Mice with epidermal loss of Notch1 as well as *Presenilin*-deficient mice develop epidermal hyperplasia and skin cancers [14], [18]. Of note, most studies have focused on Notch1 and downstream signaling members such as Rbpj or Hes1 [19], [20]. Very little is known about the function of Notch2 and other receptors in skin physiology and carcinogenesis. Here, we investigate the role of Notch1 and Notch2 using two different *Cre* expression systems. Our results provide evidence for different roles of Notch1 and Notch2 in skin development and carcinogenesis.

## Results

### *Notch1* but not *Notch2* deletion increases susceptibility to *Kras^G12D^* induced skin carcinogenesis in *Pdx1-Cre* mice

**Figure 1:**
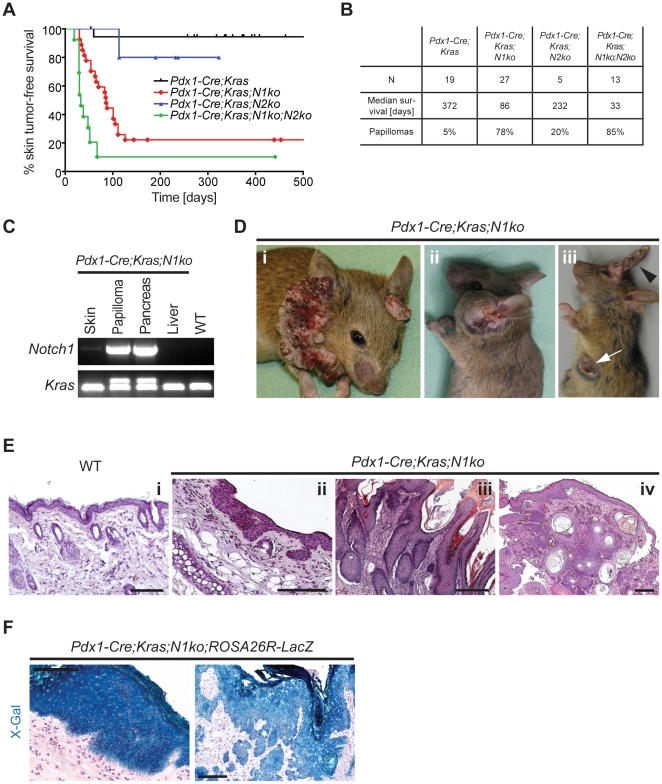
*Pdx1-Cre;Kras;N1ko* mice develop skin tumors. **A:** Kaplan-Meier tumor-free survival analysis of *Pdx1-Cre* mice. **B:** Table summarizing survival and skin tumor incidence observed in *Pdx1-Cre* mice. **C:** PCR results confirm *Notch1* deletion and *Kras^G12D^* activation in pancreas and skin papilloma while non-recombined status in unaffected skin, liver and in WT control DNA. **D:** Examples of skin neoplasia observed: papillomas of neck-head and ear (i), sebaceous gland tumor (ii), cutaneous horns (iii, black arrowhead) and SCC (iii, white arrow). **E:** Hematoxilin and eosin staining (HE) of WT skin (i) and characteristic cutaneous histopathologies found in *Pdx1-Cre;Kras;N1ko mice*: hyperplasia (ii), skin papilloma (iii) and SCC (iv). **F:** X-Gal staining indicates Cre-mediated recombination in skin hyperplasia (left) and papillomas (right) of *Pdx1-Cre;Kras;N1ko*;*ROSA26R-LacZ* reporter mice. The scale bars represent 50 µm.

To analyze the effect of *Notch1* and *Notch2* deficiency during pancreas carcinogenesis, we crossed previously described *Pdx1-Cre* [2], *Notch1^fl/fl^* [21], *Notch2^fl/fl^* [22] and *Kras^+/LSL-G12D^* [3] mice for generation of *Pdx1-Cre;Kras^+/LSL-G12D^*, *Pdx1-Cre;Kras^+/LSL-G12D^*;*Notch1^fl/fl^* and *Pdx1-Cre*;*Kras^+/LSL-G12D^*;*Notch2^fl/fl^* mice (referred to as *Pdx1-Cre;Kras*, *Pdx1-Cre;Kras;N1ko* and *Pdx1-Cre;Kras;N2ko*, respectively). These mice were born at the expected Mendelian ratio and successful recombination of the floxed loci in the pancreas was confirmed by PCR (Fig. 1C). Surprisingly, *Pdx1-Cre;Kras;N1ko* mice developed focal skin hyperplasia at 10–15 days of age and as early as 4 weeks of age developed massive skin papillomas (Fig. 1D). These lesions and tumors showed recombination of the floxed loci (Fig. 1C) pointing to epidermal *Cre* expression, which was further corroborated using *Pdx1-Cre;Kras;N1ko;ROSA26R-LacZ* reporter mice (Fig. 1F) [23]. The penetrance of the skin papilloma development was 78%. In contrast, *Pdx1-Cre;Kras;N2ko* mice rarely developed any skin phenotype. However, double *Notch1* and *Notch2* knockout mice (*Pdx1-Cre;Kras;N1ko;N2ko*) featured an accelerated skin tumor formation (Fig. 1A and B) suggesting an essential role of Notch1 ablation in epidermal lesion development and a promoting role of Notch2 deletion. *Pdx1-Cre;Kras* mice manifested a skin phenotype with low penetrance, which has been observed previously [3], [24]. Most tumors encountered in *Pdx1-Cre;Kras;N1ko* mice were benign papillomas but often grew large and ulcerating, requiring euthanasia of animals for ethical reasons. Hence, the intended pancreatic carcinogenesis study was inconclusive (data not shown).

*Pdx1-Cre;Kras;N1ko* mice developed the following skin pathologies: squamous papillomas involving the ear, neck, lips, anal and vulvo-vaginal skin, epidermal cysts, and sebaceous gland hyperplasia and cutaneous horns to lesser extend (Fig. 1D and E). Moreover, 32% of the animals developed squamous cell carcinomas (SCC), (Fig. 1E), supporting the previous observations that papillomas progressing to SCC are a common manifestation of activated Ras signaling [25]–[27]. Mice without oncogenic *Kras^G12D^* but ablation of *Notch1* and *Notch2* (*Pdx1-Cre;N1ko*, *Pdx1-Cre;N2ko*) only very rarely developed skin abnormalities (not shown).

### Evidence of *Pdx1* expression *in vivo* and *in vitro*

**Figure 2:**
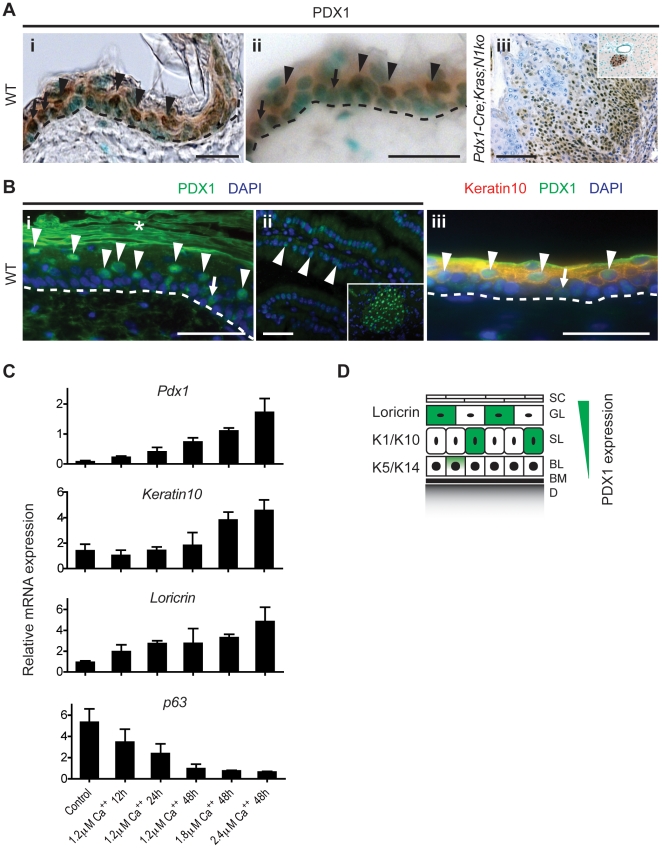
Pdx1 is physiologically expressed in the adult mouse epidermis. **A:** Immunohistochemical PDX1 staining of normal wildtype epidermis (i, ii) reveals that PDX1 is expressed in suprabasal keratinocytes (black arrowheads) and only rarely in basal cells (black arrows). *Pdx1-Cre;Kras;N1ko* papilloma (iii) is strongly positive for PDX1. Inclusion (iii) shows positive staining of pancreatic islet cells. Nuclei were contrastained with methyl green (i, ii) or hematoxilin (iii). **B:** Immunofluorescent PDX1 staining (i) indicates positive keratinocytes in the suprabasal (white arrowheads) and the basal (arrow) layer of the skin. Signal strength is comparable to that in duodenum cells (ii, arrowheads) and weaker than in pancreatic islet cells (ii, inclusion). Double immunofluorescence (iii) demonstrates that the majority of PDX1^+^ cells co-localize with a suprabasal marker Keratin10 (arrowheads) however, a small subset of PDX1^+^ cells can be found in the basal layer of the epidermis (arrow). Asterisks indicate unspecific staining of stratum corneum. **C:** Pdx1 expression in cultured keratinocytes is increased during Ca^++^-induced differentiation. Quantitative RT-PCR of *Pdx1*, *Keratin10*, *Loricrin* and *p63* transcripts in induced primary keratinocytes *in vitro*. **D:** Schematic representation of PDX1 expression in the epidermal layers: (SC) Stratum Corneum, (GL) Granular Layer, (SL) Spinous Layer, (BL) Basal Layer, (BM) Basement Membrane, (D) Dermis and their markers: Loricrin, K1/10, K5/14. The scale bars represent 50 µm.

The observation that *Pdx1-Cre;Kras;N1ko* mice develop skin neoplastic lesions with high penetrance and undergo Cre-mediated recombination are evidence of *Cre* expression in the epidermis possibly due to *Pdx1-Cre* transgene misexpression or physiological PDX1 expression in the skin. To test both hypotheses, immunohistochemical expression analysis was performed in the skin of wildtype and *Pdx1-Cre* mice, which showed a small subset of PDX1^+^ cells (Fig. 2A). Thus, the observed phenotype is due to physiological PDX1 expression in the skin rather than transgenic misexpression of Cre recombinase.

Immunofluorescent staining of PDX1 shows that the intensity of staining was comparable to that in the duodenum and much lower than in pancreatic islet cells (Fig. 2Bi and ii). Double immunofluorescent staining revealed that PDX1 co-localizes with Keratin10 (K10) in the spinous layer of the epidermis (Fig. 2Biii; arrowheads). Noteworthy, a very small fraction of PDX1^+^ cells was located in the basal layer of the epidermis suggesting that PDX1 expression may be initiated also in this layer (Fig. 2Bi and iii; arrows).

Above-mentioned experiments demonstrate that PDX1 is predominantly present in differentiated keratinocytes of the skin. To test whether PDX1 expression is regulated during keratinocyte differentiation we induced terminal differentiation in cultured wildtype keratinocytes by calcium as described [28]. As early as 12 hours after calcium addition growth arrest and a switch in keratin expression occurred. As expected, treated keratinocytes showed a three-fold induction of the differentiation markers *Keratin10* and *Loricrin* and a five fold reduction of *p63* associated with amplifying keratinocytes in the basal layer of the epidermis. In addition, we found a robust 10-fold induction of *Pdx1* transcript expression in treated keratinocytes (Fig. 2C). These findings strongly support the hypothesis that *Pdx1* is predominantly expressed in suprabasal layers of the epidermis (Fig. 2D).

### Mosaic epidermal *Cre* expression in *Pdx1-Cre* mice

**Figure 3:**
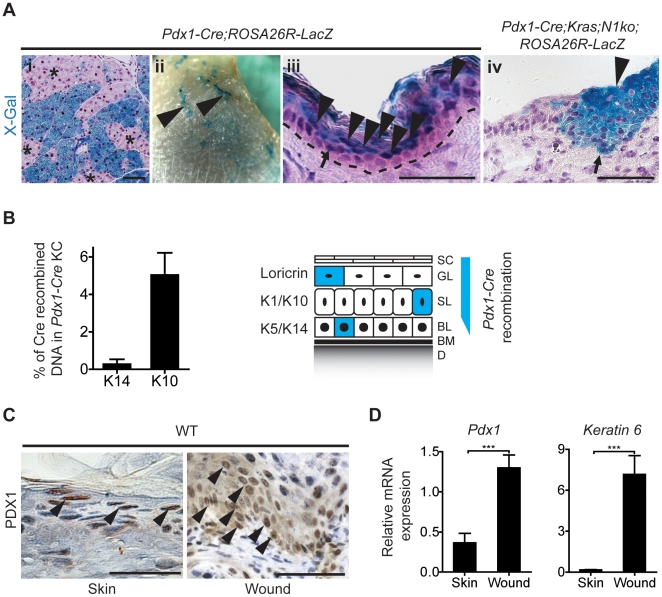
Mosaic Cre-mediated recombination in *Pdx1-Cre* mice. **A:** *Pdx1-Cre;ROSA26R-LacZ* reporter mice reveal patchy X-Gal staining as surrogate for the Pdx1 cell lineage in the pancreas (i) and in whole mount skin (ii). Analysis of X-Gal^+^ areas of the epidermis indicates that recombined keratinocytes are localized primarily in suprabasal layers of the skin (iii). Early cutaneous hyperplasia sections demonstrate that X-Gal^+^ cells are also located in the basal layer of the epidermis (iv). Asterisks indicate non-recombined areas of pancreatic tissue; arrowheads point to recombined X-Gal^+^ cells and regions; arrows show positive basal layer keratinocytes. **B:** Cre*-*mediated recombination of the *Notch1* locus occurs predominantly in suprabasal keratinocytes (K10^+^) with a small fraction of recombined basal cells (K14^+^). Schematic depiction of areas of possible *Pdx1-Cre* driven recombination in the epidermis (right): (SC) Stratum Corneum, (GL) Granular Layer, (SL) Spinous Layer, (BL) Basal Layer, (BM) Basement Membrane, (D) Dermis, (KC) Keratinocytes. **C:** Immunohistochemical staining of healing wound epidermis indicates increased expression of PDX1 in keratinocytes comparing to normal skin. **D:** Expression of *Pdx1* along with *Keratin6* is induced in wounded skin as revealed by qRT-PCR. The scale bars represent 50 µm.

Physiological PDX1 expression in the epidermis does not explain the stochastic character of papilloma formation in the *Pdx1-Cre;Kras,N1ko* mice. Hence, we speculated that *Cre* expression has a mosaic character or alternatively may be induced by mechanical skin irritation. To address the first hypothesis we examined X-Gal expression in *Pdx1-Cre;ROSA26R-LacZ* reporter mice [23]. Consistent with previous studies, we found that *Pdx1-Cre* mice showed a mosaic recombination pattern in the pancreas [1] (Fig. 3Ai). Interestingly, similar mosaic staining was observed in the skin (Fig. 3Aii). Microscopic evaluation of X-Gal positive areas indicated that suprabasal keratinocytes underwent recombination (Fig. 3Aiii; arrowheads), supporting the hypothesis that PDX1 is mainly expressed in differentiated keratinocytes. However, we found sporadically X-Gal^+^ keratinocytes residing in the basal layer (Fig. 3Aiii; arrow). All examined skin hyperplasia had X-Gal^+^ basal layer cells suggesting that neoplastic structures originate from the basal keratinocytes of the skin (Fig. 3Aiv; arrow).

To further asses the scale of recombination in the basal layer (K14^+^) and the spinous layer (K10^+^) of the epidermis we tested freshly isolated keratinocytes from *Pdx1-Cre;N1ko* mice. Cells were fractioned for K14 and K10 expression respectively using fluorescent activated cell sorting (FACS). Cre-mediated recombination was measured using quantitative PCR amplifying the recombined allele of floxed *Notch1* that was normalized to input and then compared to fully recombined DNA. We found that only 5% of DNA isolated from total keratinocytes underwent recombination in *Pdx-Cre;N1ko* mice and most of them were found in the suprabasal layer. We sporadically (below 0.5%) found K14^+^ cells with recombined Notch1 loci hypothesizing that these cells could be the cell-of-origin for papilloma development (Fig. 3B).

As papilloma development in *Pdx1-Cre* mice usually occurred in regions susceptible to grooming, scratching and wounding, we speculated that PDX1 expression may be induced in wounded skin triggering Cre-mediated *Kras^G12D^* activation and *Notch1* ablation. To test this hypothesis, wounds were induced on the back skin of wild type mice. Six days after wound formation mice were sacrificed and sections of scared skin were dissected and analyzed. Increased PDX1 expression was found in the scar tissue and in the transition zone between normal and wounded epidermis (Fig. 3C). PDX1 staining pattern was nuclear and partially cytoplasmic as previously described [29]–[32]. Quantitative RT-PCR indicated a three-fold induction of *Pdx1* and highly increased *Keratin6* transcript levels in wounded compared to normal epidermis (Fig. 3D) supporting PDX1 expression in wounded skin. In summary these results denote (i) physiological *Pdx1* expression in the skin, (ii) restricted to differentiated keratinocytes but sporadically present in K14^+^ basal cells, (iii) mosaic *Pdx1-Cre* epidermal expression, and (iv) *Pdx1* induction in wounded skin.

### Histopathology of skin tumors developing in *Pdx1-Cre;Kras;N1ko* mice

**Figure 4:**
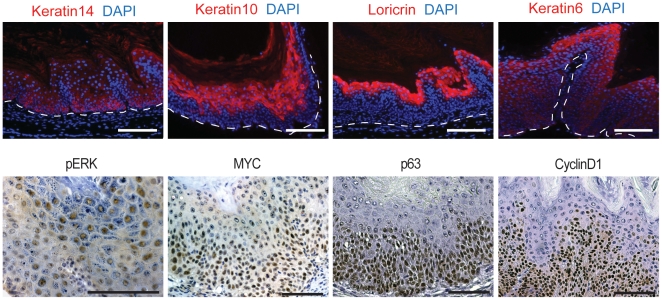
Characterization of papillomas developing in *Pdx1-Cre;Kras;N1ko* mice. Keratin14, Keratin10 and Loricrin expression show well-differentiated stratified squamous neoplasia. Keratin6 expression indicates pathological growth (upper panel). Immunohistochemical analysis of commonly activated pathways and markers expressed in *Pdx1-Cre;Kras;N1ko* papillomas (lower panel). The scale bars represent 50 µm.

Histological investigations revealed that the papillomas and hyperplastic epithelium cover thin expansions of a fibroblastic stroma often with mild chronic inflammatory infiltrates. Local hyperplasia and squamous papillomas were well differentiated, rarely demonstrating focal dysplasia (Fig. 1E). Sections of typical papillomas were analyzed by immunofluorescence for differentiation markers including Keratin 14, 10 and Loricrin. In the papillomas all three keratins were expressed in a manner similar to normal skin, except that there was a delay in the onset of K10 expression consistent with an expansion of the proliferative compartment expressing K14 and CyclinD1 (Fig. 4). In line with the hyperplastic character was the expression of K6, a keratin usually expressed in hair follicles or in pathological conditions resulting in hyperplasia (Fig. 4). The observed keratin expression pattern is characteristic of well-differentiated squamous papillomas. Older mice developed hyperproliferative lesions that exhibited cellular atypia, increased mitosis and an invasive growth pattern with characteristic keratin ‘pearls’ formation and a high degree of keratinization that are diagnostic of well-differentiated SCC. Of note, no basal cell carcinomas (BCC) were observed in *Pdx1-Cre;Kras;N1ko* mice and no signs of a metastatic disease were observed.

Immunohistochemical characterization of papillomas revealed strong activation of Ras-dependent phospho-ERK consistent with previous studies [33] as well as robust MYC expression associated with skin neoplastic transformation [34]. Interestingly, robust p63 expression throughout the papilloma tissue was noted. Normally, the presence of p63 is restricted to the thin layer of basal keratinocytes due to inhibition by Notch1. Expression of p63 is characteristic for progenitor and multiplying cells of the epidermis. Expanded and strong CyclinD1 staining supports this conclusion (Fig. 4). This expression pattern is common and characteristic for cutaneous neoplasia.

### Notch1 but not Notch2 is a tumor suppressor in the skin

**Figure 5:**
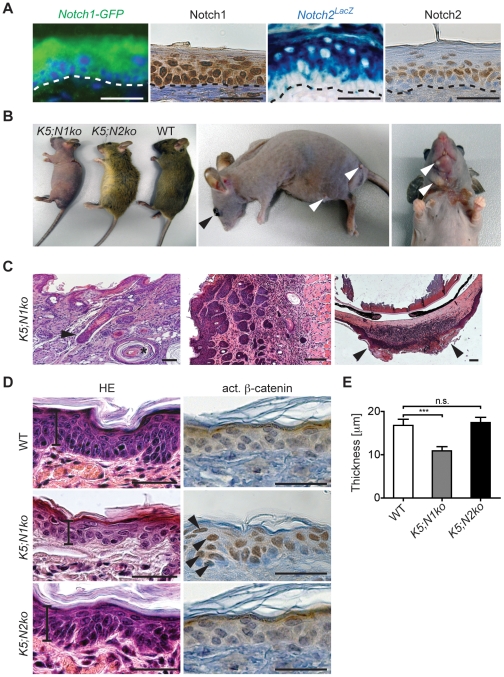
Phenotype of *K5;N1ko* and *K5;N2ko* mice. **A:** Notch1 is expressed in all layers of the adult skin whereas Notch2 is expressed only in the suprabasal layer as assessed using immunohistochemical staining and *Notch1-GFP* and *Notch2^LacZ^* reporter mice. **B:** Gross phenotype of *K5;N1ko*, *K5;N2ko* and WT mice at 4 weeks of age (left). Spontaneous skin tumors (white arrows) and hyperplastic opaque corneas (black arrowhead) start to develop in 9 months old *K5;N1ko* mice (middle and right). **C:** Skin histopathologies of *K5;N1ko* mice include epidermal cyst (asterisk), hair follicle malformation (black arrowhead, left), skin tumors (middle), hyperplasia of the cornea (black arrows, right). **D:** HE stain shows morphology and thickness (indicated by scale lines) of WT, *K5;N1ko*, *K5;N2ko* epidermis (left panel). Immunohistochemical staining reveals ubiquitous expression of active β-catenin in *K5;N1ko* (black arrows) comparing to WT and *K5;N2ko* mice epidermis (right panel). **E:** The thickness of *K5;N1ko* epidermis is significantly reduced compared to *K5;N2ko* and WT. The scale bars represent 50 µm.

**Figure 6:**
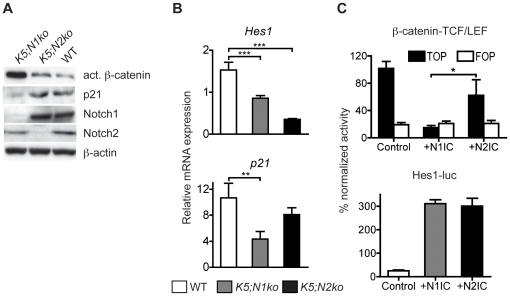
Biochemical analysis of *K5;N1ko* and *K5;N2ko* keratinocytes. **A:** Western blot analysis of primary keratinocytes isolated from different genotypes indicates correct *Notch1* and *Notch2* ablation and shows distinct modulation of β-catenin signaling and p21 expression. **B:** Quantitative RT-PCR show *Hes1* and *p21* transcripts levels in primary keratinocytes of the indicated genotypes. **C:** Luciferase reporter assay reveals that N1IC is a more potent inhibitor of β-catenin-LEF/TCF-sensitive TOP plasmid than N2IC. FOP plasmid is β-catenin-LEF/TCF-insensitive and serves as a specificity control. Both N1IC and N2IC induce Hes1 in a comparable manner as quantified using a Hes1-luc reporter.

Although the role of Notch receptors in the skin has already been intensively studied [12]–[17], we aimed to characterize epidermal *Notch1* and *Notch2* deficiency in our model. To do so, *Notch1^fl/fl^* [21] and *Notch2^fl/fl^* [22] mice were crossed with basal keratinocyte-specific *Keratin5-Cre* mice [35] (named *K5;N1ko* and *K5;N2ko* respectively). These mice were born at the expected Mendelian ratio (Fig. 5B) and successful recombination of the floxed loci was confirmed in isolated primary keratinocytes by immunoblot (Fig. 6A).

Consistent with previous studies, *K5;N1ko* mice did not develop proper hair follicles showing a ‘naked’ phenotype. Additionally, the epidermis was thinner, easily cracking and prone to injury (Fig. 5B, D and E). Such a phenotype has been attributed to a role of Notch1 in the stimulation of keratinocyte differentiation [19], [36], [37]. Before the age of 9 months, *K5;N1ko* mice developed extensive hyperplasia and keratinization of the corneal epithelium, which resulted in opaque plague formation and blindness (Fig. 5B and C) [14]. All analyzed mice (n = 4) developed skin neoplasia at 9 to 12 months of age and additionally BCC, SCC and papillomas were noticed (Fig. 5B and C). By contrast, *K5;N2ko* mice featured a non-pathological skin and hair follicle formation (Fig. 5B and D) with normal growth cycles. However, impairment of hair growth direction that manifested in more upwards-ruffle appearance of fur was observed (Fig. 5B). Mice followed up to 12 months of age (n = 4) did not show any sign of tumorigenesis. Taken together, our findings confer that Notch1, but not Notch2 is a tumor suppressor and plays a crucial role in proper skin development and differentiation.

Since expression in different compartments may explain distinct Notch1 and Notch2 functions, we analyzed the expression pattern of these receptors using immunohistochemical staining as well as transgenic *Notch1-GFP* [38] and *Notch2^lacZ^* knockin [39] reporter mice. We found Notch2 and X-Gal as a surrogate for Notch2 expression in spinous and granular layers of the epidermis (Fig. 5A). Notch1 and GFP expression in *Notch1-GFP* mice was found throughout the epidermal layers as previously described [37], including the basal layer of keratinocytes formed by stem cells and highly proliferative transit amplifying cells (Fig. 5A). Besides these differences in expression, different and context-specific functions of Notch1 and Notch2 have been described. We thus isolated and cultured primary keratinocytes from *K5;N1ko* and *K5;N2ko* mice, which showed no protein expression of the respective Notch receptor (Fig. 6A) and significantly downregulated levels of *Hes1* transcripts (Fig. 6B)

Notch1 signaling is essential for proper skin differentiation through induction of p21 (WAF1/Cip1) [37], [40]. We speculated that Notch2 signaling might not be required for this process since it is expressed mainly by differentiated keratinocytes. p21 is a cyclin-dependent kinase inhibitor that induces cell cycle arrest [41], predictably its loss is commonly associated with skin malignancies, particularly in an active Ras context [34]. We found that p21 expression was highly reduced in Notch1 ablated cells whereas no significant differences were noted in *Notch2* deficient keratinocytes both on mRNA and protein level (Fig. 6A, B). These results support the hypothesis that p21 is mainly regulated by Notch1 but not by Notch2 potentially due to cell- and context-specific differences.

### Notch1 but not Notch2 is a suppressor of β-catenin in the skin

As an increased level of active β-catenin is commonly associated with skin malignancies [18], [42], [43], we investigated the regulation of this pathway in *Notch1* and *Notch2* ablated epidermis. Immunohistochemical analysis revealed increased levels of nuclear localized β-catenin (active β-catenin) in *K5;N1ko* mice in agreement with previous studies [14]. Remarkably, neither wildtype nor *K5;N2ko* mice showed strong epidermal active β-catenin staining (Fig. 5D). Furthermore, immunoblot analysis of primary keratinocytes isolated from *K5;N1ko and K5;N2ko* mice exhibited a similar pattern (Fig. 6A).

Differences in expression of Notch1 and Notch2 in the epidermal layers as well as receptor-specific regulatory mechanisms may contribute to distinct and potentially tumorigenic alterations of β-catenin activity. Therefore, we examined the capabilities of active Notch1 (N1IC) and Notch2 (N2IC) to inhibit β-catenin signaling activity in primary keratinocytes using a luciferase reporter assay. Both Notch receptors were able to inhibit β-catenin activity but N1IC was a significantly stronger inhibitor. Forced expression of N1IC represses β-catenin signaling by over 90% whereas N2IC overexpression leads only to a modest reduction of 30% (Fig. 6C). At the same time both Notch receptors showed a similar induction of *Hes1* promoter activity, serving as a read-out for similar activation of canonical Notch signaling (Fig.6 C).

Taken together, these results support a context- and cell-specific function in addition to a distinct expression pattern of Notch and Notch2 in keratinocytes.

## Discussion

Neoplasms originating from cutaneous epithelial cells are the most common cancer-type in the United States with an annual incidence of over 1 million cases [44]. Developmental signaling pathways play a key role in the induction and progression of cancer. Our study reports a previously unrecognized epidermal expression of PDX1 and adds further evidence for a pivotal role of Notch1 but not Notch2 as a tumor suppressor in the skin, which may be particularly interesting in the light of new therapeutic approaches targeting single Notch receptors [45], [46].

### Epidermal PDX1 expression

As PDX1 is mainly expressed in the pancreas and duodenum, the *Pdx1* promoter is commonly utilized for pancreas-specific transgenic mouse lines. Surprisingly, we found conditional gene deletion in the skin using a *Pdx1-Cre* strain [2]. Further research provided strong evidence that PDX1 is physiologically expressed in the suprabasal layers of the skin (Fig. 2A and B; arrowheads) and rarely in basal keratinocytes (Fig. 2A and B; arrows). A similar pattern of *Pdx1* expression was observed in differentiation induced cultured keratinocytes (Fig. 2C). This hypothesis is supported by reports indicating a skin phenotype of *Pdx1* knockout mice, which survive 6.5 days postpartum and have, among other characteristic features, thin and cracking skin with little or no fur [7]. While these skin abnormalities may be due to indirect effects, they suggest a role of PDX1 during skin development, which should be addressed in further studies, e.g. by analyzing keratinocyte-specific *Pdx1* knockout mice, which however is beyond the scope of this report.

**Figure 7:**
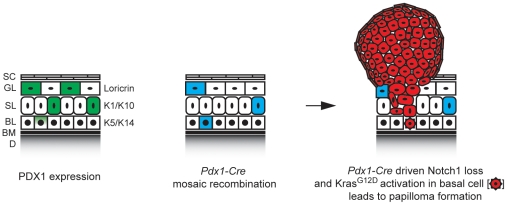
Model of epidermal Pdx1 expression and Cre-mediated epidermal recombination. Recombination rarely occurs in basal layer keratinocytes but leads to papilloma formation in *Pdx1-Cre;Kras;N1ko* mice: (SC) Stratum Corneum, (GL) Granular Layer, (SL) Spinous Layer, (BL) Basal Layer, (BM) Basement Membrane, (D) Dermis.

In contrast to the ubiquitous expression of Pdx1 in the suprabasal layers of the skin, *Pdx1-Cre;Kras,N1ko* mice developed skin papillomas and other cutaneous lesions only in preferred sites suggesting that Cre-mediated recombination may be mosaic and/or occurs in the cells resistant to neoplastic transformation. Notably, *Cre* expression in *Pdx1-Cre* mice is mosaic such that Cre-mediated recombination occurs far less frequently as would be suggested by the observed PDX1 expression. In addition, papillomas and most other skin tumors typically originate from the basal layer; in fact development from the suprabasal layer is a rather unlikely scenario (Fig. 7). Although PDX1 is mainly expressed in the suprabasal keratinocytes, we occasionally found PDX1 expression and Cre-mediated recombination in K14^+^ cells (Fig. 3A, B and 7). These observations may be the reason for the relatively few tumors developing per animal. Interestingly, tumors of *Pdx1-Cre;Kras,N1ko* mice usually develop around exposed areas of the skin (Fig. 1D), possibly due to *Pdx1* activation in wound and scar associated basal layer keratinocytes (Fig. 3C). We speculate that cutaneous aggravation or micro-wounds due to grooming and scratching may trigger an inflammatory reaction and wound healing processes with upregulated Pdx1 and Notch expression [47], thus forming a tumor-prone environment in *Pdx1-Cre;Kras;N1ko* mice.

Intriguingly, other studies have reported skin phenotypes using *Pdx1-Cre* mice despite the fact that different transgenic strains were utilized [3], [24]. These reports support our finding that *Pdx1* is expressed in the skin. However, only defined genetic alterations lead to a cutaneous phenotype. In the most often analyzed *Pdx1-Cre;Kras* mouse model, skin lesions were only rarely observed (below 5%, Fig.1B and [3], [24]). In our study, *Pdx1-Cre;Kras;N1ko* but not *Pdx1-Cre;Kras;N2ko* or *Pdx1-Cre;Kras* developed skin lesions (Fig. 1A and B) which points to the importance of Notch1 but not Notch2 for skin tumor development.

### Notch1 and Notch2 play different roles in skin tumorigenesis

Different Notch receptors have often distinct expression patterns, ligand preferences and discrete downstream signaling. Although different Notch receptors can compensate each other e.g. in pancreas development [48], individual Notch receptors commonly have distinct functions in development [49], tumorigenesis [46], [50]–[52] or tissue regeneration [53]. The result of this study points to differences in expression pattern and distinctive cellular effectors as main cause of the diverse Notch1 and Notch2 knockout phenotypes. First, we found that Notch1 and Notch2 are present only in partially overlapping layers of the epidermis. Consistent with previous studies, Notch1 is present throughout all skin layers including the tumor-prone basal layer of the skin, whereas Notch2 is expressed exclusively in suprabasal keratinocytes [37]. These findings were confirmed using immunohistochemical staining as well as *Notch1-GFP* and *Notch2^LacZ^* reporter mice (Fig. 5A). This divergent expression pattern is very likely at least partially responsible for the downregulation of p21 in *Notch1*- but not *Notch2*-deficient keratinocytes and in line with previous studies [37], [40]. p21 is a cyclin-dependent kinase inhibitor that induces cell cycle arrest [35] and its loss is commonly associated with skin malignancies, particularly in an active Ras context [36]. In *Kras^G12D^*-induced tumorigenesis inhibition of p21 *via* Myc activation, observed in *Pdx1-Cre;Kras;N1ko* papillomas (Fig. 4), is a critical step for malignant transformation [34]. Thus, the observed differences in p21 induction by Notch1 and 2 receptors (Fig. 6A and B) could partially explain the observed phenotypes.

The second notable difference between Notch1 and Notch2 was their ability to inhibit β-catenin-mediated signaling. β-catenin is responsible for hair-follicle morphogenesis and epidermal stem cell maintenance [54], whereas the disruption of the β-catenin signaling has been associated with several malignancies of the skin [18], [42], [43]. *Notch1* deficiency leading to accumulation of β-catenin in the nucleus has been associated with tumorigenesis [14]. Surprisingly, we did not observe a similar effect when the *Notch2* receptor was abrogated (Fig. 5D and 6A). Additionally, we provide *in vitro* evidence of different inhibition capacities between both receptors (Fig. 6C) further supporting the postulate of distinct molecular functions of Notch1 and Notch2.

In line with the non-redundant roles of Notch1 and Notch2 in keratinocytes is the accelerated papilloma formation in double *Notch1/2*-deficient mice (Fig. 1A and B), suggesting that Notch2 cannot fully compensate for Notch1 loss. Besides different roles in regulation of p21 and β-catenin, Notch expression dosage may play a role as was recently shown [17]. In this study *Notch1* loss promoted skin tumorigenesis in a non-cell autonomous manner by impairing skin-barrier integrity and creating a wound-like microenvironment in the epidermis. Of note, *Notch2* ablation alone had no such capabilities unless combined with a *Notch3* knockout, suggesting that a certain threshold of Notch signaling is essential for skin homeostasis.

In conclusion, our results provide strong evidence for epidermal expression of *Pdx1* as of yet not identified function as well as distinctive roles of Notch1 and Notch2 in skin tumorigenesis potentially *via* different p21 and β-catenin pathway modulation.

## Materials and Methods

### Mouse strains

*Kras^+/LSL-G12D^*, *Notch1^fl/fl^*, *Notch2^fl/fl^*, *Pdx1-Cre* and *Keratin5-Cre* transgenic mice have been described before [2], [21], [22], [27], [35]. Mice were interbred to obtain *Pdx1-Cre;Kras^+/LSL-G12D^* (*Pdx1-Cre;Kras*), *Pdx1-Cre*;*Kras^+/LSL-G12D^*;*Notch1^fl/fl^* (*Pdx1-Cre;Kras;N1ko*), *Pdx1-Cre;Kras^+/LSL-G12D^;Notch2^fl/fl^* (*Pdx1-Cre;Kras;N1ko*), Keratin5-Cre;*Notch1^fl/fl^* (*K5N1ko*) and Keratin5-Cre;*Notch2^fl/fl^* (*K5N2ko*) mice. Previously described reporter strains *LSL-ROSA26R-LacZ*, *Notch1-GFP* and *Notch2^lacZ^* [23], [38], [39], [55], were used as indicated in the text. All animals were of mixed C57BL/6J;129SV background. Animal care and experimental protocols were conducted in accordance with German animal protection laws and approved by the Institutional Animal Care and Use Committee at the Technical University of Munich.

### Statistical Analyses

Kaplan-Meier curves were calculated using the tumor free survival time for each mouse from all littermate groups. The log-rank test was used to test for significant differences between the four groups. For gene expression analysis the unpaired two-tailed *Student's* t-test was used. For *P* values the following scale was used: * *p*<0.05, ** *p*<0.01, *** *p*<0.001.

### Histology and Immunohistology

For morphologic, immunohistochemical, and immunofluorescence studies specimens were fixed in 4% buffered formalin then processed as described previously [56] and embedded in paraffin. Tissues were sectioned 4 mm and stained with hematoxylin and eosin (HE) or used for immunohistochemical studies with antibodies: CDK4 (Santa Cruz Biotechnology), K14, K10, K6, Loricrin (Covance), Notch1 (Abcam), Notch2 (The Developmental Studies Hybridoma Bank), pERK, (Cell Signaling), p63, CyclinD1 (BD), active-β-catenin (Upstate), PDX1 (gift of C.V. Wright). X-Gal staining of cryosections (10 mm) was carried out according to standard protocol, counterstained with nuclear fast red. Immunofluorescence was performed using Alexa 488 and 555 (Invitrogen). Nuclei were stained with DAPI. Pictures were taken using an Axiovert 200 M fluorescence inverse microscope equipped with the Axiovision software (Zeiss).

### Histopathological Evaluation

HE stained sections were evaluated by a pathologist (B.S.) with expertise in human and mouse cancer pathology. The pathologist, where needed, also reviewed immunohistochemical stainings.

### Western Blot Analysis

Protein extracts from freshly isolated primary keratinocyte cells were obtained using RIPA buffer containing proteinase inhibitors - Complete (Roche). Lysates were separated on standard SDS-PAGE electrophoresis, transferred to PDVF membranes as described previously [56] and incubated with antibodies: β-actin (Sigma), Notch1 (BD Pharmigen), Notch2 (The Developmental Studies Hybridoma Bank), p21 (LabVison), active β-catenin (Upstate). Antibody binding was visualized using horseradish peroxidase-labeled secondary antibodies and ECL reagent (Amersham).

### Primary Keratinocytes Culture

Keratinocytes were isolated from 3 to 4 week old mice as described previously [57]. Briefly, mice in anlagen phase were sacrificed, trunk skin was removed disinfected and enzymatically treated to allow separation of epidermis from dermis. Detaching keratinocytes were collected, filtered through Teflon mesh (100 µm), washed and plated on Petri dish previously coated with collagen and fibronectin. Cells were maintained in DMEM Spiner modification media (Sigma) with addition of 8% FCS treated with Chelex (BioRad), 10 µg/ml Transferrin, 5 µg/ml Insulin, 10 µM Phosphoethyloamine, 10 µM Ethyloamine, 0.05 nM CaCl_2_ (Sigma), 10 ng EGF, 0.36 µg/ml Hydrocortisone (Chemicon), 1% Glutathion, 1% Pen/Strep (Invitrogen).

Keratinocytes were plated and cultured for 3 to 5 days before use in luciferase and differentiation assays. Growth medium was changed every day. Induction of keratinocyte differentiation was achieved by addition of CaCl_2_ to final concentration of 1.2–2 µM.

### Fluorescent Activated Cell Sorting for Cre-mediated recombination analysis in Keratinocytes

Total isolated keratinocytes were stained with K14 or K10 antibodies (Covance) for 1 h at 4°C. Cells were washed in PBS +1% BSA and stained with the secondary antibody Alexa 488 (Invitrogen). Keratinocytes were washed and stained with propidium iodide followed by sorting using a FACS Aria 2 (BD Bioscience). DNA was isolated from the sorted cells utilizing DNeasy Blood & Tissue Kit (Qiagen) following the manufacturer's instructions. Recombination of genomic DNA was quantified by qPCR using the following program: 95°C for 10 min, 35 cycles of 95°C for 10 sec, 62°C for 10 sec and 72°C for 30 sec on a LightCycler 480 (Roche). All samples were analyzed in triplicate. β-globin genomic fragment was used for normalization. The following primers were used:

β-globin-F 5′-CCAATCTGCTCACACAGGATAGAGAGGGCAGG-3′

β-globin-R 5′-CCTTGAGGCTGTCCAAGTGATTCAGGCCATCG-3′

Del Notch1-F 5′-TGT GCT TTC ACA CTG GCA CAG-3′

Del Notch1-R 5′-CCA CTT AGA AGG AAT TCC ACC-3′

### Luciferase assay

A luciferase reporter assay was performed with a pair of luciferase reporter constructs TOPFLASH, containing three copies of the TCF/LEF binding sites and FOP-FLASH, containing mutated binding sites (Upstate Biotechnology). Primary keratinocytes were cultured in 6-well plates and transiently transfected in triplicates with Fugene 6 (Roche) and TOP/FOP or Hes1-luc plasmids with addition of forced expressing active Notch1 (N1IC) or Notch2 (N2IC) pcDNA3 plasmids and pRL-TK (Promega). Luciferase activity was measured with the Dual-luciferase reporter assay system (Promega), with the Renilla luciferase (pRL-TK) activity as an internal control, 48 h after transfection. The experiment was repeated three times, the mean of all results was taken and expressed as a percentage of induction over control ( = 100%).

### Wounding and preparation of wound tissue

Skin wound healing analysis was performed as described previously [58]. Briefly, full-thickness excisional skin wounds (6 mm in diameter) were made in WT mice. Animals were killed 5 days after wounding (*n* = 4), and an 8–10 mm area, including the complete epithelial margins, was collected and used for histopathological analysis. Three small areas (3×3 mm) of wounded and unaffected skin from the same animal were used to prepare RNA for expression analysis. Four mice were analyzed.

### Quantitative RT-PCR

RNA was isolated from primary keratinocytes using Qiagen RNeasy Isolation Kit followed by cDNA synthesis (SuperScript II, Invitrogen). Real-Time PCR was performed with 800 nM primers diluted in a final volume of 20 µl in SYBR Green Reaction Mix (Applied Biosystems). RT-PCRs were performed as follows: 95°C for 10 min, 45 cycles of 95°C for 10 sec, 60°C for 10 sec and 72°C for 10 sec. using LightCycler 480 (Roche). All samples were analyzed in triplicate. Cyclophilin and HPRT were used for normalization. The following primers were used:

K6a-F 5′-GAGCTGGCTTTGGTGGTG-3′

K6a-R 5′-GTCCTCCACTGTGTCCTG-3′

K10-F 5′-GCCAGAACGCCGAGTACCAACAAC-3′

K10-R 5′-GTCACCTCCTCAATAATCGTCCTG-3′

Loricrin-F 5′-TCACTCACCCTTCCTGGTGC-3′

Loricrin-R 5′-CACCGCCGCCAGAGGTCTTC-3′

Hes1-F 5′-AAAATTCCTCCTCCCCGGTG-3′

Hes1-R 5′-TTTGGTTTGTCCGGTGTCG-3′

p21-F 5′-CACAGCGATATCCAGACATTCAG-3′

p21-R 5′-CGGAACAGGTCGGACATCA-3′

Pdx1-F 5′-TGCCACCATGAACAGTGAGG-3′

Pdx1-R 5′-GGAATGCGCACGGGTC-3′

Cyclophillin-F 5′-ATGGTCAACCCCACCGTGT-3′

Cyclophillin-R 5′-TTCTGCTGTCTTTGGAACTTTGTC-3′

Hprt-F 5′-GACCGGTCCCGTCATGC-3′

Hprt-R 5′-CATAACCTGGTTCATCATCGCTAA-3′
